# Assessment of Antifouling Potential of Novel Transparent Sol Gel Coatings for Application in the Marine Environment

**DOI:** 10.3390/molecules24162983

**Published:** 2019-08-16

**Authors:** Chloe Richards, Ciprian Briciu-Burghina, Matthew R. Jacobs, Alan Barrett, Fiona Regan

**Affiliations:** School of Chemical Sciences and DCU Water Institute, Dublin City University, Glasnevin, Dublin 9, Ireland

**Keywords:** sol gel chemistry, hydrophobic, hydrophilic, antifouling, biofilm formation, transparent coatings

## Abstract

In recent years, there has become a growing need for the development of antifouling technology for application in the marine environment. The accumulation of large quantities of biomass on these surfaces cause substantial economic burdens within the marine industry, or adversely impact the performance of sensor technologies. Here, we present a study of transparent coatings with potential for applications on sensors or devices with optical windows. The focus of the study is on the abundance and diversity of biofouling organisms that accumulate on glass panels coated with novel transparent or opaque organically modified silicate (ORMOSIL) coatings. The diatom assessment was used to determine the effectiveness of the coatings against biofouling. Test panels were deployed in a marine environment in Galway Bay for durations of nine and thirteen months to examine differences in biofilm formation in both microfouling and macrofouling conditions. The most effective coating is one which consists of precursor, tetraethyl orthosilicate (HC006) that has a water contact angle > 100, without significant roughness (43.52 nm). However, improved roughness and wettability of a second coating, diethoxydimethylsilane (DMDEOS), showed real promise in relation to macrofouling reduction.

## 1. Introduction

**Figure 1:**
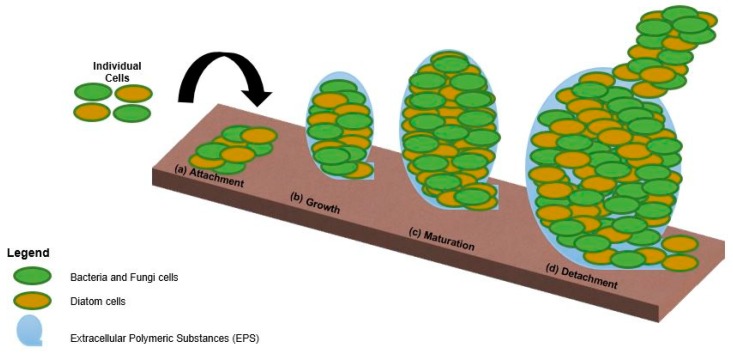
Primary steps in biofilm formation and dispersal (from left to right). (**a**) Attachment; (**b**) growth; (**c**) maturation and (**d**) detachment [5].

Biofouling is often defined as the build-up of biologically derived organic matter on artificial surfaces immersed in an aquatic environment [1]. Biofilm formation is often complex and occurs in several steps as illustrated in Figure 1. The process generally begins with the transportation of bacteria and organic molecules towards a submerged surface. From this, the conditioning layer is formed which involves the adsorption of organic molecules (i.e., proteins, carbohydrates and nucleic acids) to the surface. This boundary layer acts as an interface between the submerged surface and bacteria providing favorable conditions for the attachment of organisms [2,3]. The next step in biofilm formation is the attachment of cells to a surface. In the case of the biofilm, a solid-liquid interface can provide an excellent environment for cells to grow as the constant flow of water past these surfaces provides fresh nutrients necessary for growth. Hydrophilic surfaces in particular have been shown to exhibit excellent antifouling capabilities (well hydrated, neutral or weakly negative, protein repellant) [4], providing an optimum environment for cell adhesion, which improves the ability of cells to grow and proliferate [5]. After this attachment step, cell proliferation leads to the formation of a micro-colony, and cellular chemical signaling activates biofilm genes which enhance the ability of micro-colonies to form and adhere to solid interfaces. Mature biofilms can then detach from the solid interfaces to facilitate the multiplication and dispersal of cells and biofilm microcolonies [5].

Fouling organisms are often divided into two classes on the basis of size; (a) microfoulers and (b) macrofoulers [6]. Microfoulers include microbial organisms such as diatoms and bacteria, while macrofoulers include larger organisms such as barnacles, polycheates and macroalgae [7,8]. Microfouling formation is made up of two steps, primary and secondary colonization. Primary colonization is a reversible process whereby diatoms and bacteria adsorb onto a conditioning layer. The build-up of primary colonizers can lead to the formation of an extracellular polysaccharide layer (EPS) that contains cells, proteins, extracellular DNA and other organic species. The main foulers in the secondary colonization include macroalgal spores and protozoa [6].

Biofouling causes huge problems in the marine environment and often is considered a burden in ocean monitoring whereby materials must be placed underwater. To date, there have been many attempts to prevent fouling on instrumentation but very little have been tested in situ on oceanographic systems for long periods of time [9]. Problems associated with fouling on sensors include the long-term quality of measurements, disturbing the biological and chemical properties of the site being studied and fluctuations in readings as a result of macro-algae blocking the optical path of the sensor [9]. Current technologies to combat fouling include the use of commercial paints. However, in 2008, regulations were put in place to insist upon a change from self-polishing copolymer organotin systems (due to toxicity in the environment) to silicone and copper-consisting antifouling technologies. The attachment growth of microfouling communities that contribute to this fouling now require in-depth understanding to provide the marine industry with the best antifouling alternatives for, sensors, AUVs and buoys [10]. Overall, biocide-based coatings are among the most widely used commercial antifouling coatings. Marine aquatic species are sensitive to biocides present in anti-fouling paints so it is important to develop environmentally-friendly coatings that prevent the adhesion of fouling organisms [11].

Sol-gel derived functional coatings are commercially available for many practical applications (including self-cleaning, anticorrosion, antireflection, etc.) [12], and are emerging as suitable non-toxic alternatives to biocidal antifouling coatings [13,14]. The sol-gel process provides mild synthetic conditions and the capacity to include inorganic and organic components at the nanometric scale. This results in coatings that are able to provide both strength and durability typical of inorganic polymers but also a wide range of versatility typical of organic polymers [12]. The physicochemical properties and functionality of the final coating can be designed to meet various requirements by using metallic and organic precursors. Organic modified silicate (ORMOSIL) coatings [15,16,17] or metal nanoparticle doped ORMOSIL [18] are hybrid inorganic-organic silica coatings that have been shown to reduce biofouling. Sol-gel glassy coatings are transparent and can be effectively applied to submerged optical windows but also to other surfaces for self-cleaning applications [19]. Other attractive features of ORMOSIL coatings include the ease of preparation (i.e., one pot or step synthesis [20]), reactivity with a multitude of surfaces (i.e., glass, fiberglass, metal, wood etc.), low curing temperatures, prolonged chemical and physical stability and ease of application (i.e., spraying, dip coating, spin coating). There are three main strategies used in the design of antifouling coatings. Fouling-resistant coatings prevent the attachment of biofoulants to surfaces. Fouling-release treatments reduce biofoulant adhesion strength facilitating the removal of organisms from surfaces. Biocidal biofouling treatments kill organisms responsible for the formation of biofilms via the use of antimicrobials within the coating matrix [18]. Fouling release surfaces rely on reduced adhesion of biofoulants and the least favorable surface energy for bioadhesion in microfouling is between 20–25 mN m^−1^ [21]. In this region of the Baier curve, bioadhesion is minimal due to the formation of weak interactions between surface EPSs and proteins [6,14,21]. Biofouling however involves a complex biochemical process, in which microfoulers (e.g., diatoms) adhere to surfaces via a complex of hydrophilic proteins (EPS) [22] while macrofoulers (e.g., barnacles) adhere via a complex of hydrophobic proteins they secrete, crosslinked by cysteine residues [23]. For example, in a paper by Finlay et al., fewer barnacles colonized hydrophobic xerogel coatings while the removal percentage of diatoms decreased with surface wettability [16]. Similarly, the fouling release of juvenile barnacles has been achieved by using low wettability and low critical surface tension xerogel surfaces [15]. An increase in protein adsorption was noticed for superhydrophobic surfaces with micrometer scale roughness when compared with smooth surfaces, however exposure to shear stress removed a larger proportion of protein from the superhydrophobic, rough surfaces than from the smooth surfaces [24]. For the past number of years, research into antibacterial surfaces has been growing from strength to strength with the proposal of a number of new ways to create surfaces that are antifouling [25]. One method described to prevent bacteria from forming on a surface is by means of a bactericidal agent. Examples of these agents may include metals (i.e., silver), metal oxides such as ZnO, chitosan’s, peptides, antiseptics or antibiotics. The addition of nanotopography, nanomaterials and nanotechnology has also been noted to improve the functionality of bactericidal agents with a number of literature reporting on its effectiveness [26,27]. In a paper by Ivanova et al., an increase in the average killing rate of 450,000 cells/min/cm^2^ was described by using columnar nanotopography, having a bactericidal effect independent of surface chemistry. Another method proposed to reduce bacterial adhesion is the use of anti-adhesive surfaces. Such surfaces may include superhydrophobic or hydrophilic surfaces [19,24,28,29,30].

However, by combination of both of these methods, a more effective antibacterial surface can be created [25]. A paper by Ellinas et al. discusses superhydrophobic surfaces, investigating the factors influencing the antifouling nature of nanostructured surfaces. It was discovered that tailoring the chemistries, enriching the surface with bactericidal agents and providing surface topography at the micron and nanoscale level, an effective antibacterial surface could be realized, without the need for complicated synthesis procedures. These surfaces termed ‘hybrid’ for their anti-adhesive and bactericidal properties, were shown to reduce bactericidal action on both a long and short term basis [25]. Another exciting, up and coming area related to creating antifouling surfaces is the use of nanostructure geometry to enhance the mechanical strength on surfaces causing lysis of cells. A paper by Elimelech et al. reports some of the first findings of using the ‘mechano-bactericidal mode of action’. It details the fabrication of single walled carbon nanotubes termed ‘SWCNTs’ that used to damage the cell membrane of bacteria, E. coli, by direct contact, providing a response to the need for novel antibacterial materials. Their findings concluded that the bactericidal effect of these nanotubes was attributed to their high aspect ratio geometry. Another study by Chen et al., reported on thin, rigid SWCNTs that were capable of enhanced piercing to the cell membranes of gut-bacteria [31]. The marine environment poses a challenge to all deployed structures and biofouling affects all items that are deployed in the aquatic environment. In environmental sensing applications, optical sensors are impacted within hours of deployment by biofilm growth. Transparent materials used in sensing such as optical windows are prone to fouling that can adversely impact data integrity. Therefore, it is necessary to employ countermeasures to reduce or remove biofilms on optical windows.

The focus of this work is to study several novel transparent sol-gel materials, to determine their effectiveness as antifouling coatings for potential application to marine deployed sensors, camera lenses, solar panels or other related technologies. In this context, a range of sol-gel coatings with increasing water surface wettability and roughness were developed. The surface energy of antifouling coatings was altered by modification of surface chemistry, while surface topology was roughened by the incorporation of amorphous fumed silica within the sol-gel. Sol-gel coatings with a selected range of surface wettabilities and topologies were deployed into a marine system in Galway Bay for periods of nine and thirteen months. A long-term initial study in the marine environment was performed to look at the colonization of diatom species to the sol-gel coatings, based on their surface chemistries and topologies and offer a comparison of performance against industrial antifouling technologies.

## 2. Results and Discussion

### 2.1. Characterization of Developed Coatings

**Figure 2:**
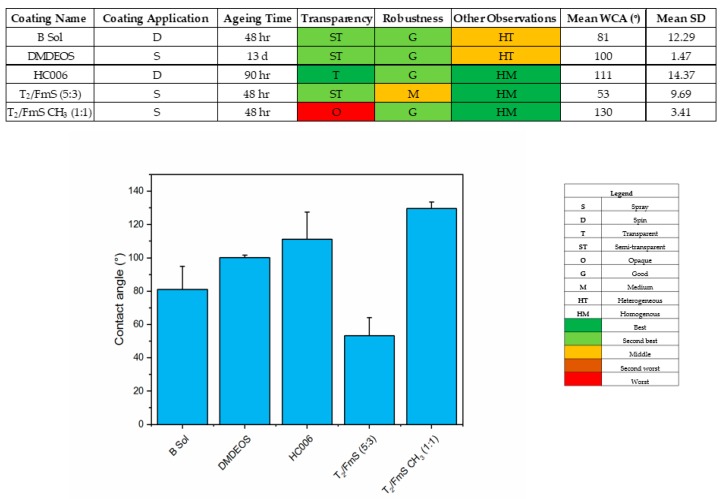
Robustness, transparency observations and water contact angle measurements for each of the sol gels used in this study (top). Water Contact Angle (WCA) measurements taken for each of the sol gel coatings before deployment in Galway Bay, Ireland (*n* = 3). Errors bars represent 95% confidence intervals.

**Figure 3:**
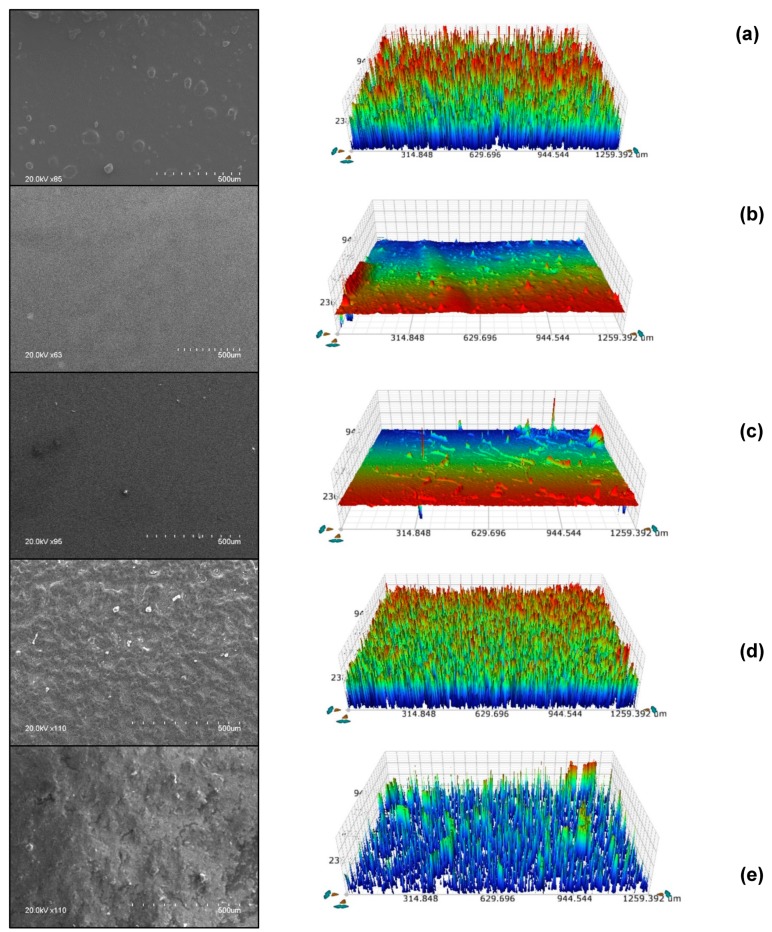
Surface characterization of the sol gel coatings with a profilometer (right) and SEM technique (left). Average surface roughness (Sa) of the coatings was recorded. (From top down (**a**–**e**); B Sol (Sa = 1624.36 nm), DMDEOS (Sa = 68 nm), HC006 (Sa = 43.52 nm), T_2_/FmS (5:3) (Sa = 846.34 nm) and T_2_/FmS CH_3_ (1:1) (Sa = 519.93 nm) (*n* = 3).

Five ORMOSIL coatings were developed and characterized prior to deployment into the marine environment in terms of robustness, transparency and water contact angle. Water contact angles (WCAs) measured on the coatings ranged from 50° to 130°, confirming the increasing wettability of the surfaces from hydrophobic to hydrophilic (Figure 2). Surface roughness measurements using white light interferometry show that a wide range of surface topographies were obtained, with average surface roughness ranging from 43 nm (HC006) to 1600 nm (B Sol) (Figure 3). SEM imaging of the coatings provides more insight into the topography and shows that smooth, homogeneous dense films were formed with sols HC006 and DMDEOS, respectively, while porous films were formed when FmS was incorporated within the sol-gel network. Coatings produced using HC006 and DMDEOS formulations generated robust, dense and fully transparent films on glass substrate. DMDEOS was previously developed in the group for self-cleaning applications and its physical characteristics (i.e., material flexibility, mechanical robustness, transparency etc.) assessed [32]. HC006 shows very similar physical characteristics with DMDEOS, with slightly higher WCAs (110°–115°). As both sols show similar average roughness values, the increase in contact angle can be attributed to a higher density of methyl (-CH_3_) groups on the surface of the coating. These groups were purposely introduced within the sol-gel by the addition of another precursor, CTMS. This precursor has only one hydrolysable group, (-Cl) and it acts as a capping agent, which terminates chain growth and adds surface functionality by lowering the surface free energy [33,34]. Furthermore, CTMS is known to have a positive effect on the mechanical properties of films by reinforcing weak points within the network and preventing drying induced cracks [35]. T_2_ sol-gel used for the preparation of T_2_/FmS CH_3_ (1:1) and T_2_/FmS (5:3) is similar in the precursor composition with HC006 (CTMS replaced with TMES and adjusted molar ratios, see methods section) but optimized to incorporate more Si-OH reactive groups. This increase reactivity was introduced to increase the adhesive potential with both the substrate and the FmS particles. The final coatings produced with this approach had an average roughness of 846 nm for T_2_/FmS (5:3) and 519 nm for T_2_/FmS CH_3_ (1:1), which is one order of magnitude higher than the average roughness of HC006 and DMDEOS. The methylation of FmS was found to have a radical effect on the wettability of the coatings, with a shift from hydrophilic (WCA = 50°) to hydrophobic (WCA = 130°), with a water rolling effect.

### 2.2. Biofouling Assessment

**Figure 4:**
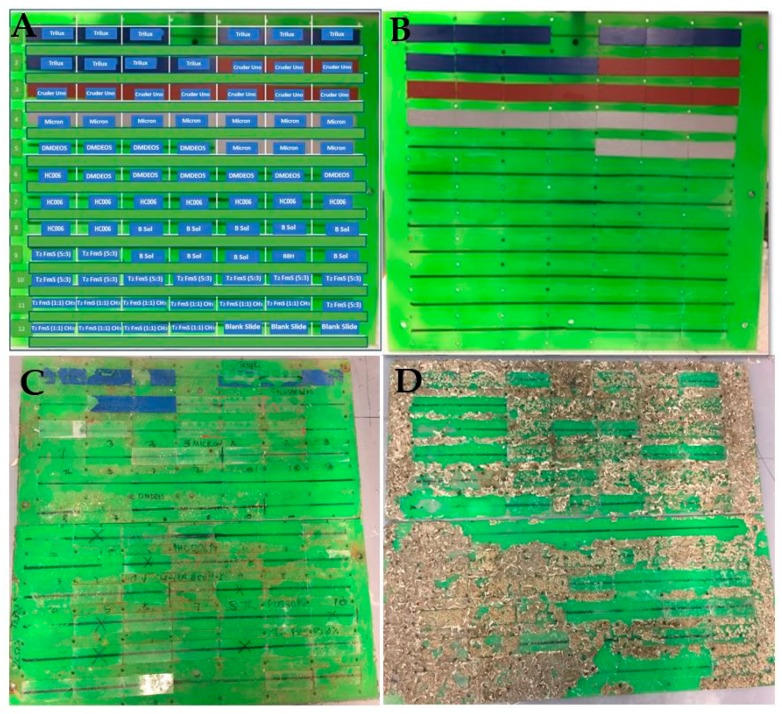
(**A**) Schematic of the layout of sol gel materials. Ten replicate slides of each type of coating were deployed (*n* = 10); (**B**) a pre-deployment clean panel; (**C**) post-deployment biofouling following a nine-month deployment from April 2017 to December 2018 during the height of the biofouling season. Depth: 2.5–3 m; (**D**) post-deployment biofouling after a 13-mo deployment from April 2017 to May 2018 including both dry and wet seasons. Depth: 24–28 m.

The effects of different sol-gel coatings on biofilm build-up were accessed. In this work, a series of novel transparent and opaque sol-gel coatings were developed, characterized and tested in the field for long periods of time. The new materials were tested alongside commercial antifouling paints. Figure 4 shows the deployment set-up, where 10 replicate slides of each material were fixed. Figure 4a shows the labelled slides, and 4b the panel before deployment. Figure 4c,d shows the panels retrieved at nine months and 13 months, respectively.

#### 2.2.1. Microfouling Assessment

As can be seen from Table 1, a wide range of fouling organisms, marine benthic diatoms, were found on the slides deployed for nine months and were identified to the species level using the scanning electron microscopy (SEM) and a diatom identification key [36]. Species diversity is a good indicator of antifouling effectiveness but is also a characteristic of the environment and time of year. It was found that there was a low species diversity overall with most species listed occurring on the slides repeatedly. Diatom species, *Cocconeis*, was found on all materials. The novel coating DMDEOS was found to be the most effective coating demonstrating minimal biofilm colonization when compared with the commercial paint, Trilux. HC006 and DMDEOS coatings were found to contain a large diversity of species of diatoms and in large numbers.

#### 2.2.2. Quantification of Protein by Lowry Assay

**Figure 5:**
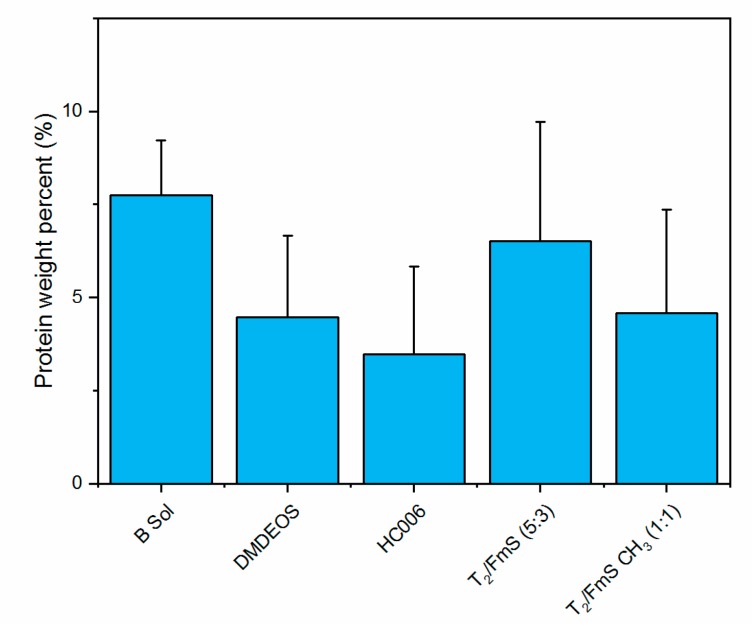
This figure displays the percent protein present on each of the sol-gel coatings deployed in the microfouling study in Galway Bay for nine months using the Lowry Protein Assay. The errors bars calculated represent 95 % confidence intervals. B Sol contained the most percent protein while HC006 contained the least.

Determination of early fouling can be done in several ways. One of these is using the biochemical assay to determine the protein associated with the exopolymeric substances associated with the initial biofilm. The results obtained from the assessment of slides retrieved after nine months deployment in Galway Bay indicate that the HC006 material has the lowest protein level, while the B sol has the greatest and is similar to T_2_/FmS (5:3) (Figure 5). The latter, however, may be an indication of non-specific adhesion rather than biofilm attachment. Protein measurement can be a better indicator of biofilm attachment due to the development of a conditioning layer during biofilm formation containing an abundance of proteins and polysaccharides and providing an optimum environment for cells to proliferate [37].

#### 2.2.3. Macrofouling Assessment

When evaluating the coatings deployed for 13-mo, it was found that macrofoulers predominate with high levels of colonization. Barnacle colonization was evident on all deployed coatings. The ANOVA significance testing was performed on the percentage coverage data collected (Excel 2016) to investigate if the commercial antifouling paints had significantly less percentage barnacle coverage than those of the sol gel coatings presented in this study. The novel transparent coating DMDEOS was found to be most effective in reducing macrofouler colonization (*p* < 0.05) long-term followed by Micron (*p* < 0.05) when compared against commercial paints and sol gel coatings when looking at percent coverage of barnacles on macrofouled glass slides.

In the formation of biofilm there are many stages as illustrated in the schematic in Figure 1. Ideally coatings will lead to reduced fouling so that deployed devices do not require frequent maintenance. For the purpose of this study, the performance of novel sol-gel coatings was compared with commercial antifouling paints.

**Figure 6:**
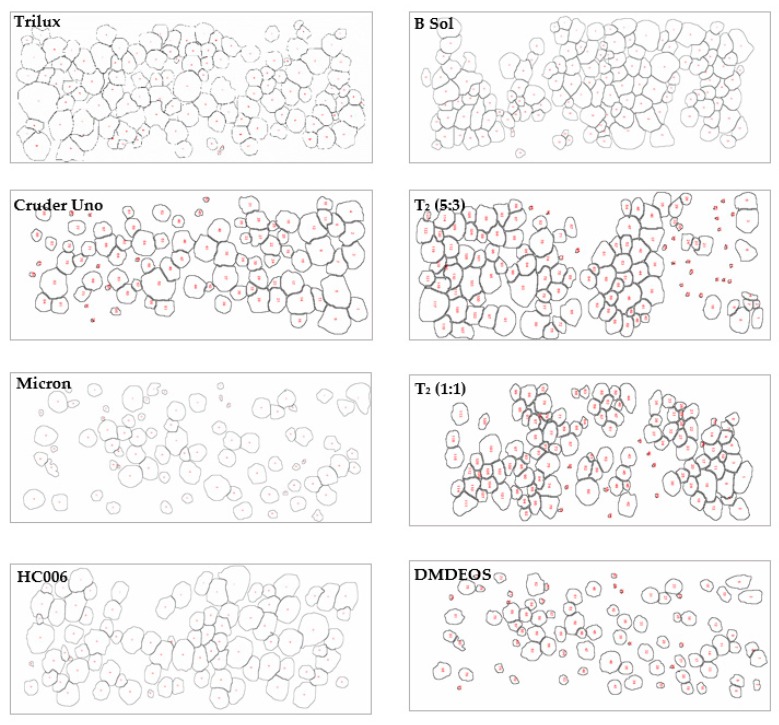
Visual data collected during the Image J analysis showing the percentage coverage of barnacles of each of the macrofouled glass slides.

Figure 6 illustrates the colonization of macrofouling organisms on materials deployed for 13 months. This table includes a subset of images of fouled materials and their analysis using the Image J software. The images are shown in Figure 6. While it is clear that all materials supported barnacle growth, the numbers are generally the same for all coatings. However, it was evident that some materials provide substrates that lead to a variety of settlement patterns. This provides useful information in understanding the chemistry and topography of effective coatings. The results indicate that the lowest coating surface coverage of 23.80%, occurs for the novel DMDEOS material, similar to commercial paint, Cruder Uno at the lowest paint surface coverage of 26.78%. The highest surface coverage observed was with the B sol at 54.15%. However, more critically, the data showing the mean nearest neighbour supports the evidence that DMDEOS is effective in reducing macrofouling colonization (higher average nearest neighbour meaning clustering of macrofouler’s is minimal), with the fumed silica sol, T_2_/FmS (5:3) demonstrating an even distribution of barnacle settlement. This information regarding spacing and clustering of fouling organisms provides valuable evidence of the benefit of the new coatings in disrupting normal settlement patterns. The effectiveness of the differing chemistries of the materials leading to more effective antifouling technologies is evident in relation to that disruption. Again, when looking at the nearest neighbour data calculated for each of the commercial paints and sols, we can see where the clustering is occurring quite clearly. The lower the nearest neighbour value given, the more clustering that is occurring. Where the nearest neighbour values have higher averages, the degree of clustering is lower and therefore fouling is reduced.

**Figure 7:**
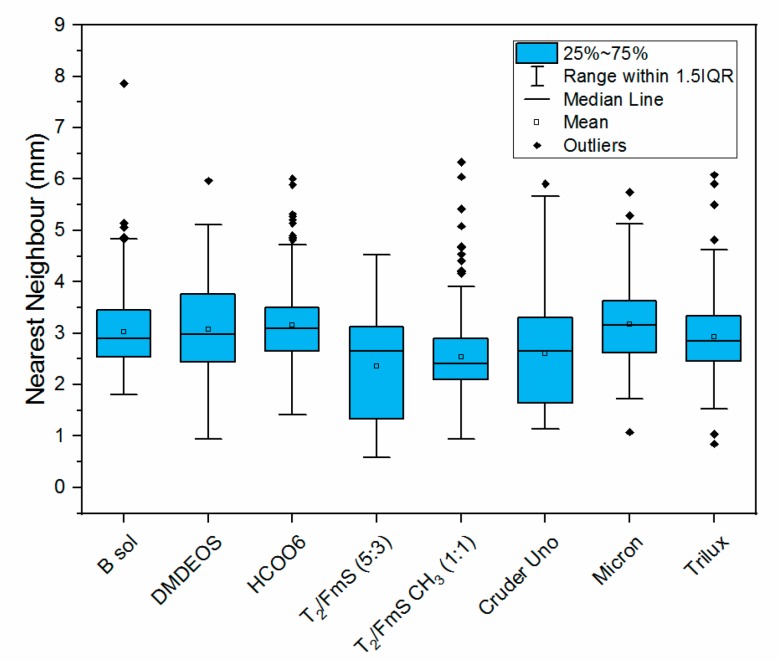
Nearest neighbour data for sol-gel coatings deployed in Galway Bay for thirteen months as part of the macrofouling study to test the overall effectiveness of our novel antifouling sol-gel coatings on a long-term basis (*n* = 3).

Figure 7 shows the detailed analysis data for determination of the nearest neighbour for each material. This data clearly shows the narrow range of values for T_2_/FmS (5:3) compared with other materials. Even the commercial paints demonstrate clustering of fouling organisms.

The average and median of each plate is shown as indicated in the figure. The bounds of the blue box show the proportion of data that is 25% above and 25% below the median of the data (i.e., the distribution of the middle 50% of the data). If the top and bottom of the blue box are dissimilar sizes this indicates that the spread of values is skewed. The interquartile range (IQR) is the size of the blue box (middle spread of the data), and the whiskers are the upper and lower half of this box either added or subtracted from the IQR multiplied by 1.5 (showing the middle 75% of the data). Errors bars represent 95% confidence intervals.

**Figure 8:**
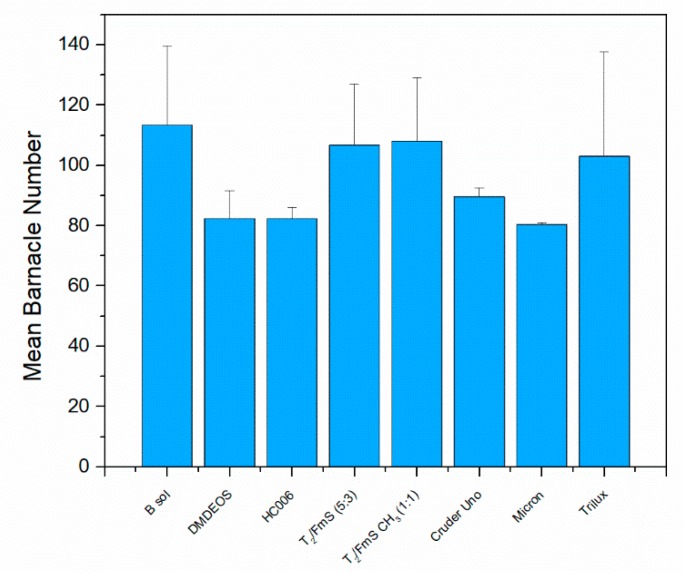
Barnacle attachment on sol-gel coatings deployed in Galway Bay for 13 months as part of the macrofouling study to test the overall effectiveness of our novel antifouling sol-gel coatings on a long-term basis (*n* = 3). Error bars represent standard deviation.

**Figure 9:**
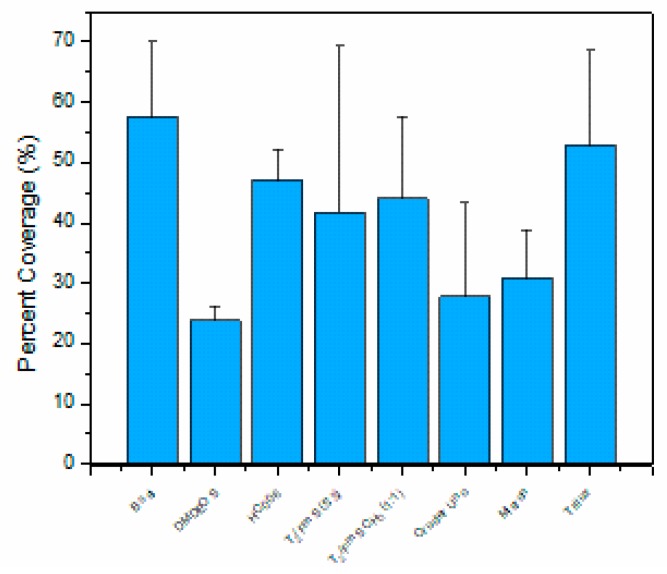
Percentage area surface coverage of barnacles attached to slides on sol-gel coatings deployed in Galway Bay for 13 months as part of the macrofouling study to test the overall effectiveness of our novel antifouling sol-gel coatings on a long-term basis (*n* = 3). Error bars represent standard deviation.

Figure 8 shows the actual barnacle density data for each deployed coating, and Figure 9 the percentage coverage of the barnacles on each of the coatings. From this assessment (Table 2) the overall settlement is similar across all materials (*p* > 0.05), but the format or pattern of adhesion is different on some materials. The most effective materials in the microfouling study arise from the T_2_ sol-gel used for the preparation of T_2_/FmS (5:3) due to the narrow diversity of diatom species observed on the glass slides. This is similar in the precursor composition with HC006 (CTMS replaced with TMES and adjusted molar ratios, see methods) but optimized to incorporate more Si-OH reactive groups. This increase in reactivity was introduced to increase the adhesive potential of the coating, with both the substrate and the FmS particles. The final coatings produced with this approach had an average roughness of 846 nm (S_ku_ = 3.094) for T_2_/FmS (5:3) which is one order of magnitude higher than the average roughness of HC006 and DMDEOS. The methylation of FmS was found to have a radical effect on the wettability of the coatings when looking at water contact angle values, with a shift from hydrophilic (WCA = 50°) to hydrophobic (WCA = 130°), with a water rolling effect. From this study, it appears that a combination of roughness and wettability is most effective in these static field studies. The most effective material in the macrofouling study when comparing the overall percentage coverage of barnacle colonization was DMDEOS (*p* < 0.05). It would be interesting to evaluate the effectiveness of this series of coatings in dynamic flow conditions in future studies.

## 3. Materials and Methods

### 3.1. Chemical Reagents 

All materials were purchased from Sigma-Aldrich (Arklow, Ireland) including Lowry reagent, Foline & Ciocalteu’s Phenol, *D*-(+)-Glucose, tetraethyl orthosilicate (TEOS), diethoxydimethylsilane (DMDEOS), ethanol, isopropanol (IPA), triethoxy(ethyl) silane (ETEOS), ethoxytrimethyl silane (TMES), chlorotrimethylsilane (CTMS), hydrochloric acid (HCl) (37%) and bovine serum albumin solution (200 mg mL^−1^) (BSA). Control paints used in this analysis include Trilux, Cruder Uno and Micron (International). All chemicals purchased were of analytical grade and aqueous solutions were prepared with deionized water (DI) (18.2 MΩ cm^−1^).

### 3.2. Instrumentation

A UV 1800 spectrophotometer (Shimadzu, USA) was used to measure the absorbance of reaction solutions in the Lowry assay for quantification of protein on of each of the sample slides. The scanning electron microscopy of coatings was carried out on a Hitachi S-3400N instrument with a 20 kV accelerating voltage. Samples were gold-coated using a 750T sputter coater (Quorum Technologies, Lewes, UK). Water contact angle (WCA) measurements were carried out using an Artray and Navitar camera with the FTA32 software. Surface roughness measurements were performed using a Contour GT profilometer (Bruker, MA, USA).

### 3.3. Preparation of Sol-Gel Coated Surfaces

**Figure 10:**
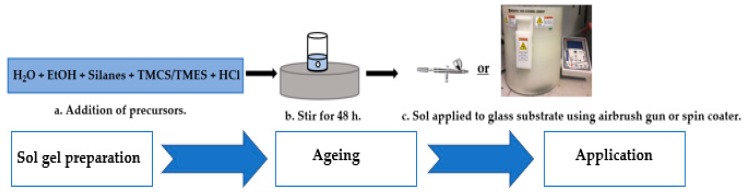
Schematic outlining procedure for sol-gel synthesis and application. Order of precursors: 1.H_2_O and EtOH; 2. Silanes; 3. TMCS/TMES; 4. HCl (37%); 5. Stir for 48 h.

A schematic representation of sol-gel synthesis and application is presented in Figure 10. Glass microscope slides (25 mm × 75 mm, Fisher Scientific, Ireland) were used as the substrate for the sol-gels and nanoparticles (NPs). Glass slides (*n* = 10) were prepared for coating via sonication in detergent and water for 30 min followed by sonication in IPA for 30 min. The glass slides were then left to dry at room temperature prior to use.

#### 3.3.1. Sol-Gels Synthesis

Sol-gels used for the generation of transparent, smooth coatings were prepared as outlined in Figure 10 using a series of silane precursor mixtures (Table 3) hydrolyzed with DI in the presence of ethanol. The sol-gels were prepared using a molar ratio of one silane: 6.25 × C_2_H_5_OH: 4 × H_2_O: 0.007 HCl. Ageing of the sol-gels to achieve condensation was allowed to proceed for 48 h—for HCOO6, T2 and B Sol and approximately 400 h—DMDEOS. Ageing was carried out at room temperature and under magnetic stirring (300 rpm).

Topography modification of the coatings was realized using amorphous fumed silica (FmS) while surface chemistry of the coating was altered by methylation of FmS using CTMS. Briefly, a 3 g quantity of FmS (Aldrich, Arklow, Ireland, 0.007 µm) was weighed and transferred to a 100 mL glass bottle. A 100 mL volume of ethanol was added to the bottle and the solution was sonicated for 30 min. After sonication, the FmS was partially methylated by the addition of chlorotrimethylsilane (CTMS). The FmS and CTMS were mixed together to give a ratio of 10:1 (*v*/*v*) FmS: CTMS. This solution was then stirred using a magnetic stirrer for 24 h at room temperature. Using the FmS dispersion and the partially methylated FmS dispersion (FmS CH_3_) two sol-gels were prepared by mixing with T_2_, i.e., T_2_/FmS CH3 (1:1) sol was prepared by mixing equal volumes of T_2_ and FmS CH_3_ (1:1, *v*/*v*) while T_2_/FmS (5:3) was prepared by mixing T_2_ and FmS in a 5:3, *v*/*v* ratio. The newly formed solutions were allowed to age for another 24 h, ambient temperature, 300 rpm.

#### 3.3.2. Application to Substrate

Glass microscope slides (Fisher Scientific, Dublin, Ireland, dimensions, 25 mm × 75 mm) were used as substrate for the deposition of sol-gel films by both spin coating and spray coating. *Spin Coating:* The DMDEOS and HC006 sol-gels were spin coated onto clean glass microscope slides at 500 rpm for 15 s using a Laurell WS-650-23 spin coater. *Spray Coating:* T_2_/FmS (5:3), T_2_/FmS CH_3_ (1:1) and B Sol sol-gels were spray coated using a WD-180R VEDA airbrush gun. Each glass substrate was placed 10 cm from the tip of the spray gun. The spray gun was then filled with the desired sol and the glass substrate was sprayed over a period of 10 s in a circular motion. Heat treatment at 110 °C was used to liberate excess solvent and stabilize the coatings.

#### 3.3.3. Characterization of Sol Gel Coatings

Water contact angle. WCA measurements were completed using an Artray and Navitar camera with the FTA32 2.0 data logging software. WCA measures the shape of ultrapure water droplets sitting on the surface of the sol-gel coatings. Each coating was made in triplicate and the WCA angle measured at three different points on each surface to calculate the mean WCA and standard deviation. *Surface roughness*. In the assessment of novel transparent antifouling coatings, it is important to consider roughness. This parameter can not only can have a significant impact on the hydrodynamic performance of an antifouling coating but can also influence the settlement pattern of biofouling organisms such as larvae [38]. Surface roughness measurements were taken in triplicate using white light interferometry on a Bruker Contour GT profilometer. Robustness. Robustness measurements were taken using a pencil hardness tester, Elcometer 501 (Elcometer inspection equipment, Warren, MI, USA). Transparency. Transparency measurements were taken using on an ocean optics spectrometer (USB2000+), a LS1 tungsten halogen lamp (ocean optics, 360 and 2000 nm emission), with a detector, QP1000-2-UV-Vis (USA) 1 mm internal diameter and optical fibres (2 m length).

### 3.4. Site Description and Deployment Procedure

**Figure 11:**
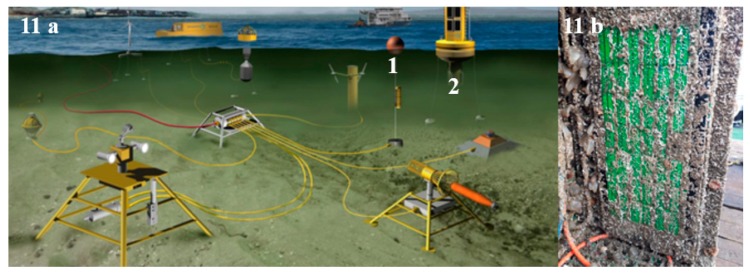
(**a**) Renewable energy test site in Galway Bay where panels were deployed. 1. Panels attached to buoy for microfouling study; 2. Panels attached to underwater observatory for macrofouling study; (**b**) right, panels deployed (Image source: Smart Bay Ireland).

Figure 11 illustrates the test bed where the materials were deployed. The location selected for this experiment was Galway Bay, County Galway, Ireland (53.2000° N, 9.2333° W), depth was approximately 2.5 m–3 m for the microfouling study (panels attached to buoy) and 24 m–28 m for the macrofouling study (panels attached to underwater observatory). Microscope slides coated with commercial paint (Trilux, Micron and Cruder Uno as control) and novel transparent antifouling coatings were deployed on a polymethyl methacrylate (PMMA) panel (57.5 cm × 25 cm) for durations of nine and thirteen months to assess the durability and performance of novel transparent coatings biofilm growth at different depths and time periods under different conditions. The deployment device containing samples was retrieved after nine and 13 months from Galway Bay by the diving team at Smart Bay Ireland. The panel containing the glass slides was fixed in Styrofoam to minimize movement and damage during transportation to the laboratory for analysis. Samples were analyzed immediately on delivery.

### 3.5. Characterization of Biofouling

#### 3.5.1. Microfouling

##### Lowry Assay for Protein Determination

Biofilm accumulated on glass slides was scraped off carefully using a cell scraper and recovered in individual sample tubes. The samples were then dissolved in 10 mL of deionized water and passed through 2.5 µm filter paper to remove large particles present in the sample.

Protein standard solutions were made up in the range of 100 µg L^−1^–400 µg L^−1^. 1 mL of each of the standard solutions made up with 200 µg mL^−1^ BSA as well as unknown samples and a blank sample were taken and placed in a sample tube. To this, 1 mL of the Lowry reagent was added. The solutions were vortexed vigorously for 1 min before being left for 20 min to react at room temperature. A 0.5 mL volume of Foline & Ciocalteu’s phenol working solution was then added to each of the samples and vortexed again to give a homogeneous solution. These solutions were then left to stand for 30 min at room temperature. This allowed for the reduction of phosphomolybdotungstate. The absorbance of each of the standard solutions, blank and unknown samples were then measured at 750 nm using deionized water used to zero the instrument.

##### Diatom Identification Using Scanning Electron Microscopy

For the identification of diatoms using SEM, a glass slide from each coating was prepared using a gold sputter coater. The samples were coated for 30 s before being analyzed under SEM. At 5 kV and 1 mm and 20 kV and 10 µm, images of the various diatom species were taken and identified at species level using a diatom identification key.

#### 3.5.2. Macrofouling

##### Image J analysis

The Image J software was used to calculate the nearest neighbour distances and macrofouling coverage in the following way; An image was taken of three replicate slides of each type of coating and loaded into the Image J software. The scale of the image was set to those of a microscope glass slide (25 mm × 75 mm). The image was converted to 8-bit and using the paintbrush tool, circles were drawn around the barnacles covering each slide. The threshold of the image was then adjusted to (0,0). The circles drawn around each of the barnacles could then be filled in using the paint bucket tool in a different colour (i.e., blue). The threshold of the image was adjusted again in the range of 160–180 (to yield an image of black circles (i.e., barnacles). The measurements were then set to include the area and centroid. The analyzed particles feature was used to calculate the number and area of barnacles present on each of the slides. Nearest neighbour distances were calculated using the plugin feature ‘nnd’ in the plugins tab of Image J [39].

##### Statistical Analysis

The ANOVA statistical analysis was performed using Microsoft Excel (2016) with the data analysis toolbar. Statistical analysis was performed to understand which sol-gel coatings were most effective at preventing macrofouler’s from attaching to glass microscope slides in comparison to commercial paints, Trilux, Cruder Uno and Micron.

## 4. Conclusions

The purpose of this study was to investigate the antifouling effectiveness of novel transparent sol-gel coatings deployed in the marine environment. Five novel materials were developed with varying chemistries leading to different hydrophobicities, wettability characteristics and roughness. The study extended greater than 12 months, with an initial evaluation of primary fouling (at nine-mo), followed by a study of macrofouling (four-mo later). It was found that materials that showed greatest diversity of organism colonization in the initial stages, did not lead to the heaviest biofilm (in terms of protein levels). However, there is no consistent pattern and further study is required. HC006 shows very similar physical characteristics with DMDEOS, with slightly higher WCAs (110° –115°). As both sols show similar average roughness values, the increase in contact angle can be attributed to a higher density of trimethylsilyl (-CH_3_) groups on the surface of the coating. Overall, the most effective coating in reducing the formation of an initial biofilm is HC006. The greatest diversity of organisms is seen, reducing the likelihood of permanent biofilm. However, this is not a very robust material and that may reduce its effectiveness over time. The material that has been most effective in reducing attachment of macrofouling organisms (barnacles) is DMDEOS. This is borne out by the overall coverage and nearest neighbour data. The roughness of the T_2_/FmS (5:3) also shows promise as an effective material worthy of further study in dynamic conditions.

A future application of this work would be to incorporate sol gel coatings on the optical windows of sensors to reduce early stage fouling. The advantage of using the sol gel method for this application lies in its ability to coat optical fibres or waveguides and precisely control sensitivity determining parameters such as film thickness and length. Depending on the method of coating, the thickness of the film can be controlled for the desired result. For example, a high film thickness is achievable using the dip coating method where the thickness can be controlled by withdrawal speed. Sol gel films exhibit excellent mechanical strength and strong adhesion properties, in particular, thin films, which display faster response times [40]. A paper by Liu et al. discusses the influence of film thickness on the detection of ammonia in water. Silica coatings of varying film thicknesses are coated on the surface of the fiber sensor. It was found that the silica coatings of higher thickness displayed a better sensitivity of 0.131 nm/ppm to ammonia than that of the thinner films (0.069 nm/ppm) [41]. Another example of the influence of sol gel film thickness on the sensor signal can be found in a paper by Mc Donagh et al. This paper involved the modification of sol gel films for enhancement of the optical sensing of O_2_ in gas and aqueous phases. It was found that the increase in film hydrophobicity occurred from the modification of the precursors within the sol gel matrix. This alteration resulted in enhanced dissolved oxygen (DO) sensor performance [42]. Clearly sol-gels with defined chemistry, roughness and wettability provide real promise as antifouling transparent coatings for sensors or other devices that require such technology.

## Figures and Tables

**Table 1:** Observed diatom settlement on coatings deployed for nine months in Galway Bay.

Sol/Genus Number *	1	2	3	4	5	6	7	8	9	10	11	12	13	14	15
B sol			+		+	+			+				+	+	
DMDEOS		+		+		+		+							
HC006	+	+	+	+	+	+	+		+	+					
T2/FmS (5:3)					+					+				+	
T2/FmS (1:1) CH_3_	+		+		+				+		+	+	+	+	+
Trilux			+		+										
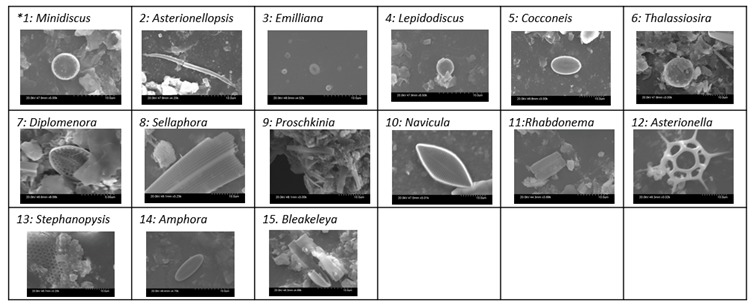															

**Table 2:** Data summary of the mean barnacle number mean surface coverage (%) and mean nearest neighbour data for the macrofouled coatings deployed for 13-mo (*n* = 3, for each sol gel). This data was measured using the Image J software (Figure 6). All coatings deployed in the macrofouling study showed barnacle and tube worm colonization only. Barnacle colonization was the only organisms accounted for in the calculation of the mean surface coverage and mean nearest neighbour data.

Material Name	Total No. of Barnacles	Mean Barnacle Area (mm^2^)	Percent Coverage (%)	Mean NN (mm)	NN SD (mm)	Mean Total Area (mm^2^)	Mean Total Area SD (mm^2^)
Trilux	309	9.63	52.91	2.93	0.75	992.11	6.32
Cruder Uno	269	5.84	26.78	2.59	1	502.05	4.71
Micron	241	7.31	30.82	4.24	2.96	577.81	5.13
HC006	247	10.66	46.81	3.16	0.72	877.65	6.16
B Sol	340	9.43	54.15	2.74	0.74	1015.32	6.99
T_2_ (5:3)	320	6.44	35.93	2.30	0.98	673.62	5.71
T_2_ (1:1)	324	6.55	37.73	2.50	0.73	707.41	5.85
DMDEOS	247	5.42	23.80	3.07	0.83	446.29	3.89

**Table 3:** Sol-gel recipes; precursors used in the synthesis of the hydrophobic transparent sol-gel coatings used for deployment in Galway Bay.

Sol Name	TEOS	ETEOS	CTMS	EtOH	HCl	H_2_O	DMDEOS	TMES
DMDEOS	2.679	-	-	18.24	0.029	3.6	6.51	-
HC006	2.763	0.886	2.094	12.04	0.007	2.378	-	-
T2	4.06	0.84	-	11.67	0.007	2.252	-	1.42
B Sol	2.793	0.886	2.094 ^1^	12.04 ^2^	0.007	2.378	-	-

^1^ Fumed silica methylated (FmS CH_3_) was used instead of ethanol (EtOH). ^2^ CTMS was added to sol after 24 h. Volumes of precursors are given in milliliters (mL).
